# Inhibiting Glutamate Activity during Consolidation Suppresses Age-Related Long-Term Memory Impairment in *Drosophila*

**DOI:** 10.1016/j.isci.2019.04.014

**Published:** 2019-04-13

**Authors:** Motomi Matsuno, Junjiro Horiuchi, Kyoko Ofusa, Tomoko Masuda, Minoru Saitoe

**Affiliations:** 1 Tokyo Metropolitan Institute of Medical Science, Setagaya, Tokyo 156-8502, Japan

**Keywords:** Behavioral Neuroscience, Molecular Neuroscience, Model Organism

## Abstract

In *Drosophila*, long-term memory (LTM) formation requires increases in glial gene expression. Klingon (Klg), a cell adhesion molecule expressed in both neurons and glia, induces expression of the glial transcription factor, Repo. However, glial signaling downstream of Repo has been unclear. Here we demonstrate that Repo increases expression of the glutamate transporter, EAAT1, and EAAT1 is required during consolidation of LTM. The expressions of Klg, Repo, and EAAT1 decrease upon aging, suggesting that age-related impairments in LTM are caused by dysfunction of the Klg-Repo-EAAT1 pathway. Supporting this idea, overexpression of Repo or EAAT1 rescues age-associated impairments in LTM. Pharmacological inhibition of glutamate activity during consolidation improves LTM in *klg* mutants and aged flies. Altogether, our results indicate that LTM formation requires glial-dependent inhibition of glutamate signaling during memory consolidation, and aging disrupts this process by inhibiting the Klg-Repo-EAAT1 pathway.

## Highlights

• Expression of the Eaat1 glutamate transporter increases during memory consolidation
• This suggests that memory consolidation requires reduced glutamate signaling
• Aged flies have reduced long-term memory and reduced Eaat1 induction
• Increasing Eaat1 or inhibiting glutamate activity improves memory in aged flies

## Graphical Abstract

**Figure:**
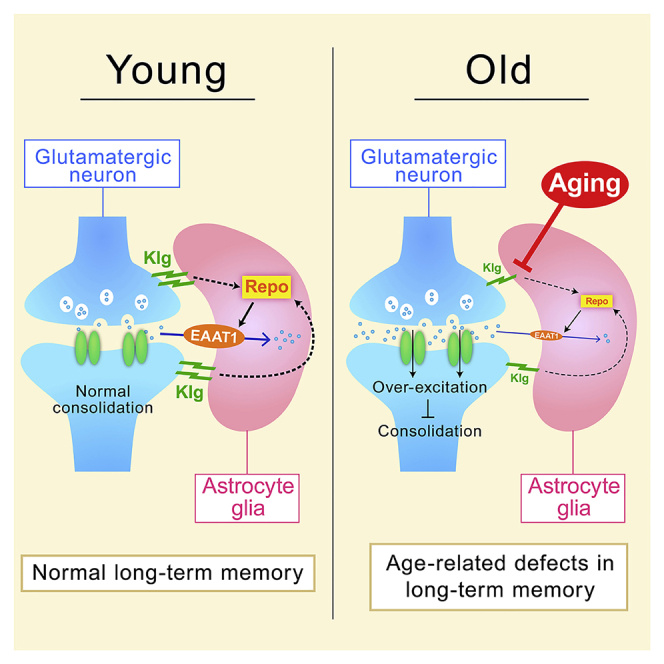

## Introduction

In many organisms including *Drosophila*, aging is associated with decreased memory, a phenomenon known as age-related memory impairment (AMI). In *Drosophila*, AMI consists of decreases in two types of memory, a short-lasting, middle-term memory (MTM), which can be measured 1 h after training (Tamura et al., 2003, Tully et al., 1990), and long-term memory (LTM), which can be measured 24 h after spaced training, repeated training with rest intervals in between trainings (Mery, 2007, Tully et al., 1994). Previous studies have identified several causes of age-related impairments in MTM (MTM-AMI), including reductions in polyamine amounts (Gupta et al., 2013), altered insulin signaling (Tanabe et al., 2017), and reductions in amounts of the neuromodulator D-serine (Yamazaki et al., 2014). However, the causes of age-related impairments in LTM (LTM-AMI) are still largely unknown (Tonoki and Davis, 2015). It is also unclear whether AMI defects in MTM are related to or independent of defects in LTM.

LTM formation requires *de novo* transcription and translation. Although transcription and translation in neurons has been relatively well studied, LTM also requires glial transcription mediated by a glia-specific transcription factor, Repo (Matsuno et al., 2015). Repo had previously been identified as a factor required for glial differentiation during development (Halter et al., 1995, Xiong et al., 1994, Yuasa et al., 2003), but its amounts also increase in adults after spaced training, and it is required acutely in astrocytic glia for LTM formation. Training-dependent increases in Repo require the cell-adhesion molecule Klingon (Klg), which is expressed in both neurons and glia and is proposed to be involved in neuron-glia communication (Matsuno et al., 2015, Matsuno et al., 2009). However, factors downstream of Repo that regulate LTM remained unexplored.

A recent report suggests that in the human brain, glial cells show larger changes in gene expression upon aging than neurons (Soreq et al., 2017). With age, glial cells lose their regional identity, whereas neuronal patterns are preserved. How these changes affect LTM-AMI has been unclear. In this study, we identify the glial glutamate transporter EAAT1 (Soustelle et al., 2002) as a downstream effector of Klg and Repo, required for LTM. We also show that the expressions of Klg, Repo, and EAAT1 decrease upon aging, and overexpression of Repo or EAAT1 restores normal LTM in aged flies. Consistent with the role of EAAT1 in reducing glutamate amounts in the synaptic cleft (Rival et al., 2004, Rival et al., 2006), we find that pharmacological inhibition of glutamate signaling after spaced training also suppresses LTM impairments in aged flies. These results indicate that glutamate signaling needs to be inhibited during memory consolidation and that age-related reductions in LTM are caused by the inability of aged glia to decrease glutamate activity during consolidation.

## Results

### Impaired Repo Expression in Aged Flies Causes Age-Related Impairments in LTM

**Figure 1:**
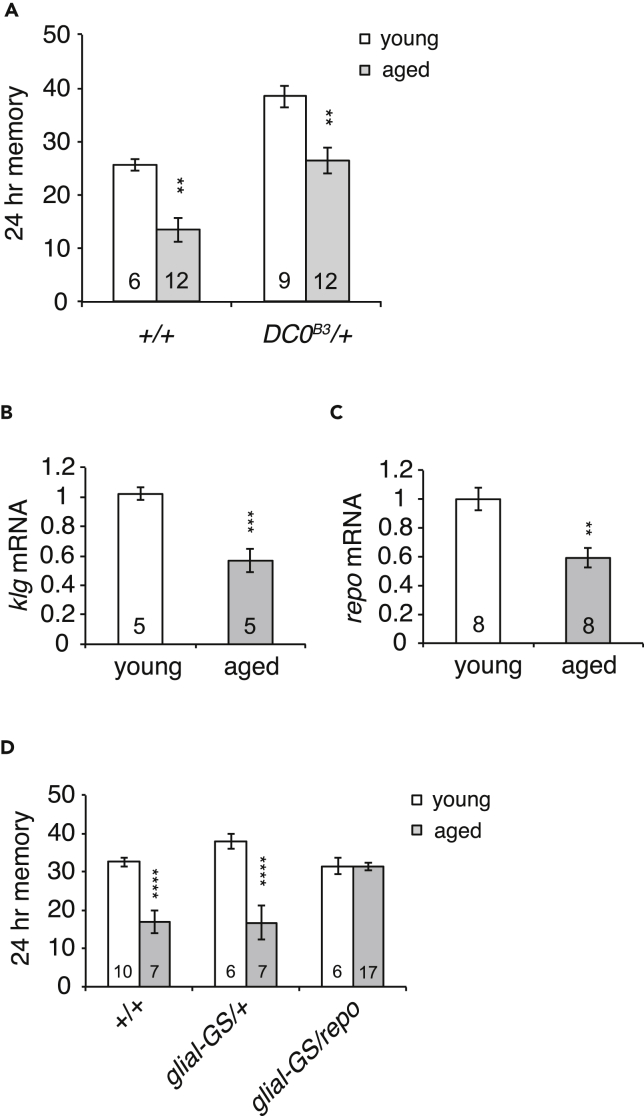
Increased Repo Expression in Glia Improves LTM in Aged Flies (A) *DC0*^*B3*^*/+* mutants show normal reductions in LTM upon aging. Wild-type flies (*+/+*) show a characteristic reduction in memory 24 h after spaced training upon aging. Three-day-old flies were used for young flies, and 25-day-old flies were used for aged flies. *DC0*^*B3*^*/+* mutants show a similar age-dependent reduction. The increased baseline memory seen in *DC0*^*B3*^*/+* mutants is due to increased ARM in these mutants. **p < 0.01 compared with appropriate young flies. Numbers in bar graphs indicate sample sizes. (B) *klg* expression decreases upon aging in wild-type fly heads as assayed by qPCR. ***p < 0.001. (C) *repo* expression decreases upon aging in wild-type fly heads as assayed by qPCR. **p < 0.01. (D) Increasing glial expression of *repo* rescues age-related impairments in LTM. *repo* was overexpressed using leaky expression from the glial-GS driver in the absence of RU486 in a *UAS-repo/+; glial-GS-GAL4/+* line. Weak overexpression was sufficient to rescue age-related impairments in LTM, whereas strong overexpression inhibited LTM in both young and aged flies (see Figure S1). ****p < 0.0001 compared with appropriate young flies. Data are represented as means ± SEMs.

Age-related impairments in MTM are ameliorated by hypomorphic mutations in *DC0*, the gene encoding the catalytic subunit of PKA in *Drosophila* (Yamazaki et al., 2007). To determine whether age-related impairments in LTM occur through a similar mechanism, we examined memory 24 h after spaced training in both young (3- to 5-day-old) and aged (25-day old) *DC0*^*B3*^*/+* flies. Memory 24 h after spaced training consists of two types, protein synthesis-dependent LTM, which decreases upon aging, and anesthesia-resistant memory (ARM), which is unaffected by aging (Mery, 2007, Tully et al., 1994; see also Isabel et al., 2004). Previously, we demonstrated that *DC0*^*B3*^*/+* mutations improve ARM in an age-independent manner (Horiuchi et al., 2008). Consistent with this, we observed a general increase in 24-h memory in both young and aged *DC0*^*B3*^*/+* mutants compared with wild-type (*+/+*) flies (Figure 1A). However, we also observed an equivalent decrease in 24-h memory upon aging in both wild-type and *DC0*^*B3*^*/+* mutants, suggesting that age-related impairments in LTM occur through a mechanism independent of impairments in MTM.

We previously demonstrated that LTM formation requires activity of Klg, a homophilic cell adhesion molecule that is expressed in both neurons and glia, and Repo, a glial transcription factor induced by spaced training. Repo activation requires Klg, indicating that Klg functions upstream of Repo in the same pathway during LTM formation. To next determine whether this Klg/Repo pathway is affected by aging, we examined *klg* and *repo* expression in heads from young and aged flies and found that expression significantly decreased at age 25 days (Figures 1B and 1C). This suggested that decreased expression may be a cause of age-related impairments in LTM, and we next examined whether age-related LTM defects could be rescued by increasing *repo* expression in aged flies using a *glial-Geneswitch-GAL4* driver (*glial-GS*) and a *UAS-repo* transgene. The *glial-Geneswitch-GAL4* driver induces expression of the *UAS-repo* transgene specifically in glial cells in the presence of an inducer, RU486. Interestingly, we found that LTM in young flies was inhibited upon strong activation of *repo* expression by feeding flies 0.5 mM RU486 for 3 days before spaced training, whereas leaky or low levels of *repo* expression in the absence of RU486 had no effects (Figure S1A). In contrast, in aged flies, LTM was unaffected by RU486-dependent repo expression and improved by leaky expression in the absence of RU486 (Figures 1D and S1C). This suggested that moderate expression of *repo* is sufficient to rescue age-related deficits in *repo* expression and LTM, whereas strong overexpression has deleterious effects. To examine this idea further, we measured Repo protein amounts in heads from young and aged wild-type flies, and from aged *glialGS > repo* flies expressing both leaky and RU486-dependent *repo* expression (Figure S1B). Aged flies showed a greater than 2-fold reduction in Repo protein amounts compared with young flies, a result consistent with the gene expression data in Figure 1C. RU486-dependent *repo* overexpression significantly increased Repo protein amounts, such that there was approximately 2-fold more Repo in aged *glialGS >repo* flies fed RU486 compared with young wild-type flies. Leaky expression, in the absence of RU486, also increased Repo in aged flies, although this increase did not reach statistical significance when compared with the amounts of Repo in aged wild-type flies. However, we note that there is also no statistical difference in Repo amounts when comparing aged *glialGS >repo* flies and young wild-type flies, suggesting that leaky *repo* expression in *glialGS >repo* flies can counter the effects of age-dependent reductions in Repo. Altogether, our results suggest that decreased Repo activity causes age-related impairments in LTM and that this impairment can be rescued by mild expression of *repo* in aged flies. We did not study the effects of *klg* on LTM-AMI because overexpression of *klg* both decreases LTM (Figure S1D) and increases lifespan (Seong et al., 2001).

### EAAT1 Is a Downstream Effector of Klg/Repo Signaling

**Figure 2:**
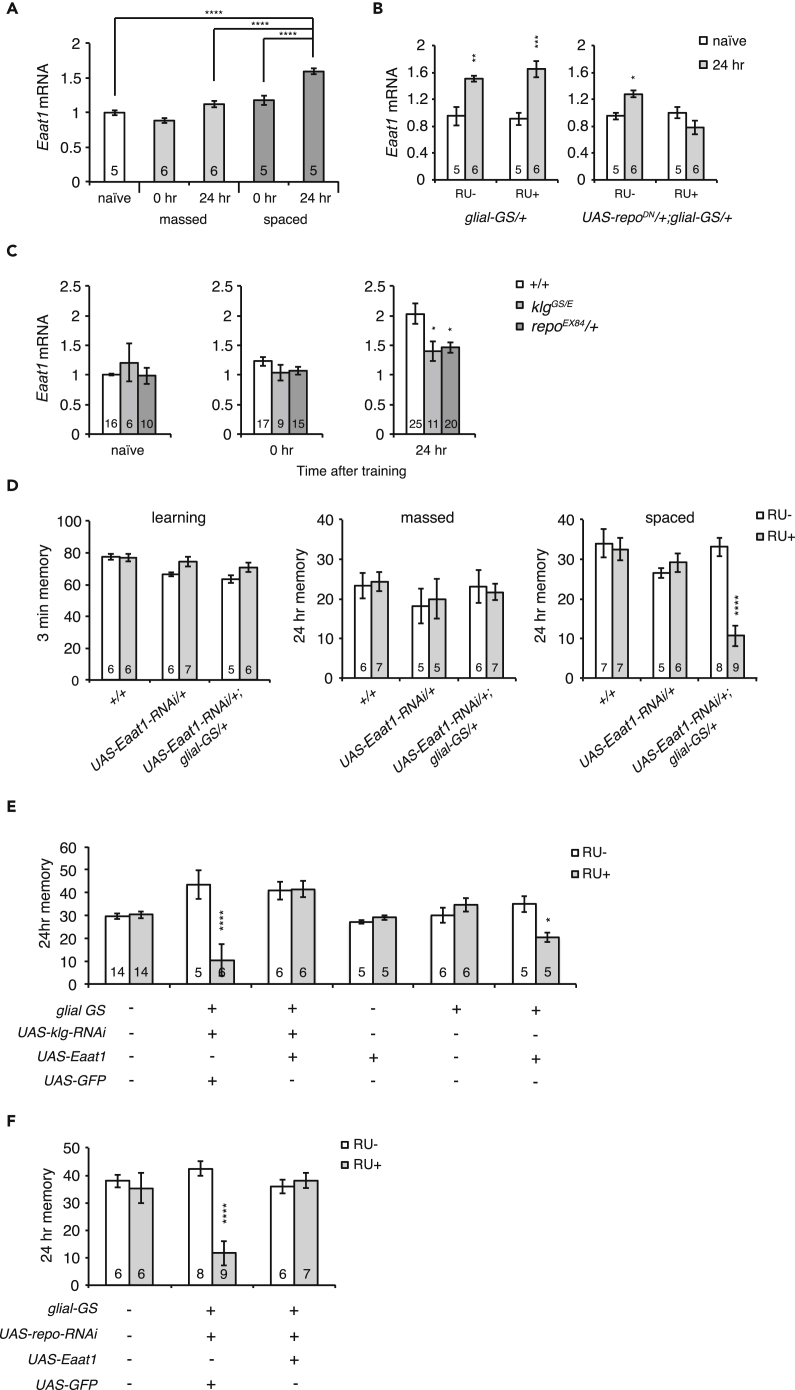
EAAT1 Is Required for LTM and Functions Downstream of Klg and Repo (A) Expression of *Eaat1* in naive and trained flies. *Eaat1* expression increased significantly 24 h after spaced training, compared with naive and massed-trained flies. One-way ANOVA indicates significant differences between samples (F_(4,22)_ = 39.19, p < 0.0001). ****p < 0.0001 compared with naive, massed-trained, and 0 h after spaced trained flies. (B) Induction of dominant negative *repo* (*repo*^*DN*^) under control of an RU-inducible glial promoter suppresses LTM-dependent increases in *Eaat1* in fly heads. RU486 (0.5 mM) was fed to flies from 3 days before training until sampling. *p < 0.05, **p < 0.01, ***p < 0.001 compared with naive flies and flies 24 h after spaced training. (C) Spaced-training-dependent increases in *Eaat1* are suppressed in *klg*^*GS/E*^ and *repo*^*EX84*^*/+* mutants. *p < 0.05 compared with wild-type flies. (D) Acute inhibition of EAAT1 specifically disrupts LTM, but not initial learning and ARM formed by massed training. The *glial-GS* driver was used to overexpress *Eaat1* RNAi upon RU feeding in a *UAS-Eaat1 RNAi/+;glial-GS-GAL4/+* line. RU486 was fed to flies from 3 days before training until testing. ****p < 0.0001 compared with RU− controls. (E and F) Glial expression of *Eaat1* rescues LTM defects of *klg* knockdown (E) and *repo* knockdown lines (F). Knockdown of *klg* or *repo* using a *glial-GS* driver (*UAS-klgRNAi/+; glial-GS-GAL4/+* RU+ or *UAS-repoRNAi, glial-GS-GAL4/+* RU+) disrupts LTM. Glial expression of *Eaat1* (*UAS-klgRNAi/+; UAS- Eaat1/glial-GS-GAL4* or *UAS-repoRNAi, glial-GS-GAL4/UAS-Eaat1*) is sufficient to rescue these defects, whereas expression of an unrelated protein, GFP (*UAS-klgRNAi/UAS-GFP; glial-GS-GAL4/+* or *UAS-GFP/+; UAS-repoRNAi, glial-GS-GAL4/+*), does not. In these experiments, we knocked down *repo* or *klg* and expressed *Eaat1* using the same *glial-GS* driver. To control for using one driver to express two different constructs, we expressed *GFP* instead of *Eaat1* in controls. See Figure S3B for *Eaat1*-dependent rescue of LTM in a *repo* mutant. *p < 0.05 and ****p < 0.0001 compared with RU− controls. Data are represented as means ± SEMs.

To understand how Repo contributes to LTM, we next searched for glial genes that are regulated by Repo and required for LTM. We used a directed screen in which we examined seven previously identified glial genes known to regulate behavior and physiology, *loco* (Granderath et al., 1999)*, axotactin* (Yuan and Ganetzky, 1999)*, Eaat1* (Soustelle et al., 2002)*, crammer* (Comas et al., 2004)*, Vdup1* (Mandalaywala et al., 2008)*, genderblind* (Grosjean et al., 2008), and *ebony* (Suh and Jackson, 2007). To determine if these genes are regulated by Repo, we measured expression in a heat-shock-inducible *repo* RNAi line. We heat-shocked *hs-GAL4/+; repoRNAi/+* flies for 30 min at 37°C 6 h before harvesting, compared expression to non-heat-shocked controls, and found that expression of *genderblind, crammer,* and *Eaat1* were significantly reduced (Figure S2A). We next examined the expression of these three genes 24 h after spaced training and found significant increases in *Eaat1* and *crammer*, but not *genderblind* (Figures 2A and S2B). As *crammer* is expressed in neurons, including the mushroom bodies (MBs), as well as glia (Comas et al., 2004), we decided to focus on *Eaat1*, which is only expressed in glia (Soustelle et al., 2002).

When we measured *Eaat1* mRNA transcripts after different training protocols, we found significant increases in expression after spaced training, which produces LTM, but not after massed training, which produces ARM (Figure 2A). *Drosophila* EAAT1 is the only high-affinity glutamate transporter in flies and is predominantly expressed in astrocyte-like glia (Rival et al., 2006). Nine Repo-binding sites are found in *Eaat1* gene region (Kerr et al., 2014), and its expression is disrupted in *repo* mutants (Soustelle et al., 2002). Thus, to next determine whether the increase in *Eaat1* expression after training occurs through Klg/Repo signaling, we examined whether this increase is prevented by a dominant-negative Repo. In *UAS-repo*^*DN*^*/+; glial-GS/+* flies, *repo*^*DN*^ is expressed in glial cells when flies are fed the drug RU486. *Eaat1* expression increased 24 h after spaced training in *glial-GS/+* control flies, and in *UAS-repo*^*DN*^*/+; glialGS/+* flies not fed RU486, whereas this increase was abolished in RU486-fed *UAS-repo*^*DN*^*/+; glialGS/+* flies (Figure 2B). Furthermore, increased *Eaat1* expression was also significantly suppressed in *klg* and *repo* mutants (Figure 2C).

To determine whether increased *Eaat1* expression is required for LTM, we knocked down the expression using two independent *Eaat1-RNAi* lines. Silencing *Eaat1* in glia did not affect learning (3-min memory), or 24-h memory after massed training, but significantly impaired 24-h memory after spaced training in *UAS-Eaat1-RNAi/+; glial-GS/+* or *UAS- Eaat1-RNAi#2/+; glial-GS/+* lines (Figures 2D and S3A). This suggests that *Eaat1* expression in the adult fly is required specifically for LTM formation.

As EAAT1 functions downstream of Klg and Repo (Figure 2C), we next examined whether defective *Eaat1* induction could be responsible for the LTM defects in *klg* and *repo* mutants. Acute silencing of *klg* or *repo* in glial cells is sufficient to inhibit LTM (Matsuno et al., 2015). Expression of an unrelated protein, GFP, has no effects, whereas we found that glial expression of *Eaat1* is sufficient to rescue these defects (Figures 2E and 2F). Furthermore, similar LTM improvement also occurs with the overexpression of *Eaat1* in a *repo*^*EX84*^ mutant background (Figure S3B). As EAAT1 clears glutamate from extracellular spaces, our results suggest that the function of glial transcription and translation is to reduce glutamate activity after spaced training during LTM consolidation.

### Defects in EAAT1 Induction Causes LTM-AMI

**Figure 3:**
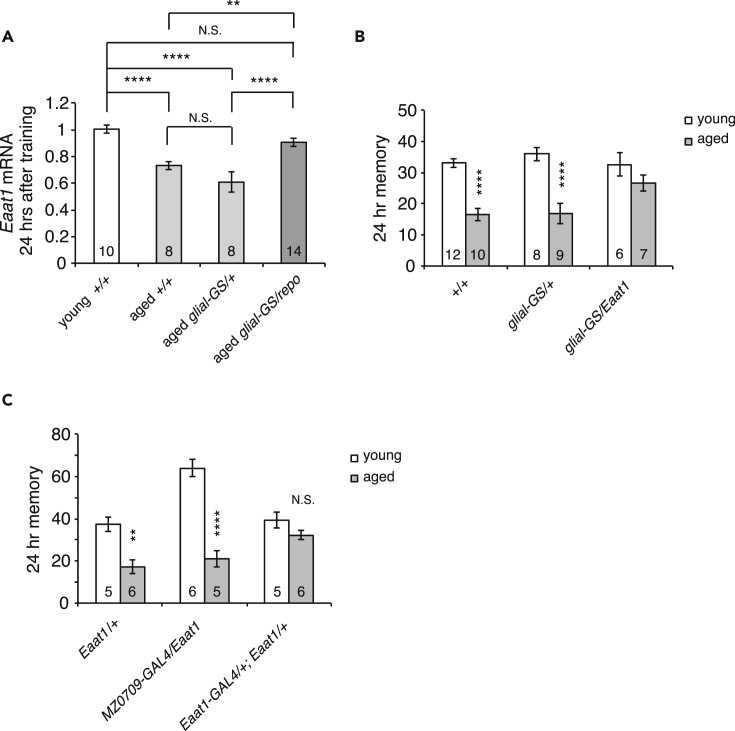
Glial Expression of EAAT1 Improves LTM in Aged Flies (A) *Eaat1* expression after spaced training is reduced in aged flies. This reduction is rescued by glial expression of *repo*. **p < 0.01, ****p < 0.0001 and N.S. as p > 0.05. (B) Glial expression of *Eaat1* rescues age-related impairments in LTM. *Eaat1* was expressed in glia using leaky expression from the *glial-GS* driver in a *UAS-repo/+; glial-GS-GAL4/+* line. ****p < 0.0001 compared with young flies. (C) Expression of *Eaat1* in astrocyte-like glia (*Eaat1-GAL4/UAS-Eaat1*) rescues age-related impairments in LTM, whereas expression in ensheathing glia (*MZ0709-GAL4/UAS- Eaat1*) does not. **p < 0.01 and ****p < 0.0001 compared with young flies. Data are represented as means ± SEMs.

As the expressions of *klg* and *repo* decrease with age, our results raised the possibility that decreased amounts of EAAT1 during consolidation may be a cause of age-related impairments in LTM. *Eaat1* expression after spaced training was significantly reduced in 25-day-old flies compared with 3-day-old flies (Figure 3A). This decrease was rescued by overexpression of *repo* in glial cells. As *repo* overexpression rescues both age-related LTM defects and decreases in *Eaat1* expression, we next examined whether *Eaat1* overexpression may be sufficient to rescue age-related LTM defects. Control flies showed decreased LTM at age 25 days, whereas *glial-GS/UAS-Eaat1* flies, which overexpress *Eaat1* in glia, maintained young memory levels at this this age (Figure 3B). This suggests that the primary cause of age-related impairments in LTM is an inability of aged flies to reduce glutamate signaling during memory consolidation.

We previously demonstrated that Repo needs to be expressed in astrocyte-like glia for LTM (Matsuno et al., 2015). EAAT1 is also expressed specifically in astrocyte-like glia. To determine the glial subtype where *Eaat1* needs to be expressed to suppress age-related impairments in LTM, we expressed *Eaat1* in both ensheathing glia (from the *MZ0709-Gal4* driver) (Doherty et al., 2009) or in astrocyte-like glia (from the *Eaat1-Gal4* driver) and measured LTM at young and old ages. When *Eaat1* was overexpressed in astrocyte-like glia, age-related impairments in LTM were significantly suppressed in aged flies (Figure 3C). In contrast, LTM was significantly reduced in aged control flies and in aged flies in which *Eaat1* was overexpressed in ensheathing glia. Thus similar to Repo, *Eaat1* needs to be expressed in astrocyte-like glia to reduce glutamate signaling during memory consolidation.

### Inhibiting Glutamate-Activity during Consolidation Rescues LTM in Aged Flies and *klg* Mutants

**Figure 4:**
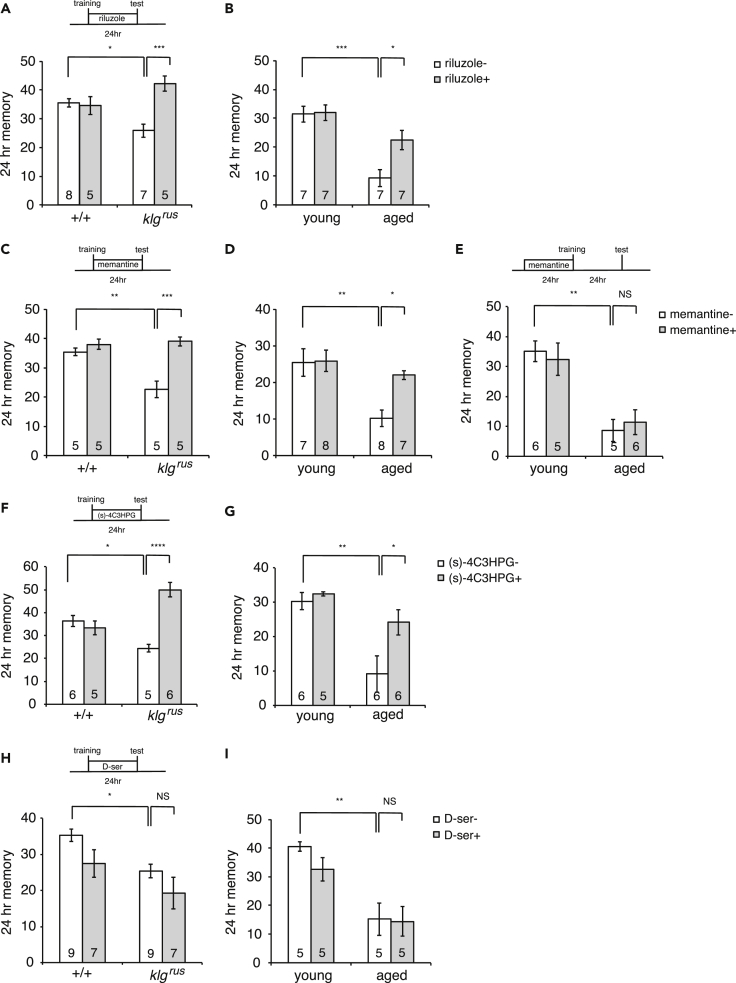
Inhibiting Glutamate Activity during Consolidation Rescues LTM in *klg* Mutants and Aged Flies (A and B) Feeding flies 1 mM riluzole, an anti-excitotoxic agent, immediately after spaced training significantly rescues the LTM in both *klg* mutants (A) and aged flies (B). *p < 0.05 and ***p < 0.001. (C and D) Feeding flies memantine (20 μg/mL), an NMDAR antagonist, after spaced training significantly rescues LTM in *klg* mutants (C) and aged flies (D). *p < 0.05, **p < 0.01, ***p < 0.001. (E) Administration of memantine before spaced training did not rescue LTM impairments in aged flies. **p < 0.01. (F and G) Feeding (s)-4C3HPG (100uM), a mGluR1 antagonist/mGluR2 agonist, after spaced training significantly rescues LTM in *klg* mutants (F) and aged flies (G). *p < 0.05, **p < 0.01, ****p < 0.0001. (H and I) Feeding D-serine (1mM), a coagonist of NMDARs, after spaced training did not rescue the LTM in either *klg* mutants (H) or aged flies (I). *p < 0.05 and **p < 0.01. In (H), two-way ANOVA indicates significant effects of D-serine feeding (F_(1,28)_ = 5.645, p = 0.0246), suggesting that increasing NMDAR activity during LTM consolidation inhibits LTM. Data are represented as means ± SEMs.

EAAT1 clears glutamate from synaptic areas, preventing neural over-excitation and glutamate toxicity (Rival et al., 2004, Rival et al., 2006). If EAAT1 works similarly during LTM consolidation, artificial inhibition of neuronal or glutamate activity may bypass the requirement for EAAT1 and restore normal LTM to *klg* mutants, *repo* mutants, and aged wild-type flies. To test this possibility, we next examined the effects of various pharmacological agents, including riluzole, an anti-excitotoxic agent (Rival et al., 2004), two NMDAR antagonists, memantine (Wang et al., 2012) and MK801 (Tomita et al., 2015, Miyashita et al., 2012), and (s)-4C3HPG, a mGluR1 antagonist/mGluR2 agonist (Zhang et al., 1999) on LTM formation. Significantly, feeding flies riluzole during the 24-h period between spaced training and testing rescued LTM defects in *klg*^*rus*^ mutants (Figure 4A) and improved LTM in aged wild-type flies (Figure 4B). Feeding flies memantine during consolidation also had similar effects, increasing LTM in *klg* and *repo* mutants and in aged wild-type flies (Figures 4C, 4D, and S4A). If aged flies were fed memantine for the same amount of time before training, LTM was not improved (Figure 4E), suggesting that glutamate activity needs to be inhibited specifically during consolidation. Another NMDA receptor (NMDAR) antagonist, MK801 also improved LTM (Figures S4B–S4D). (s)-4C3HPG had similar effects to memantine, MK801, and riluzole when fed during consolidation (Figures 4F and 4G). In contrast, feeding flies D-serine, an NMDAR coactivator that suppresses age-related impairments in 1-h memory (Yamazaki et al., 2014), did not improve LTM in *klg* mutants or in aged flies (Figures 4H and 4I). Instead, D-serine feeding during consolidation tended to attenuate LTM in young wild-type flies, suggesting that increasing NMDAR activity during consolidation may inhibit LTM. Taken together, these results suggest that EAAT1-dependent inhibition of glutamate activity is necessary for LTM consolidation and aged flies have a deficiency in this process.

## Discussion

Changes in glial transcription due to neuronal activity have been studied previously (Hasel et al., 2017, Muthukumar et al., 2014, Steward et al., 1991), but a specific role of glial transcription in LTM has been less characterized. Expression of the glial transcription factor, Repo, increases shortly after spaced training, and this increase is required for LTM formation (Matsuno et al., 2015). In this report, we identified *Eaat1* as a Repo-regulated glial gene required for LTM consolidation. *Eaat1* encodes a glial glutamate transporter that removes glutamate from synaptic sites and transports it into astrocytes (Besson et al., 1999, Kanai, 1997, Takahashi et al., 1997). Thus, our data indicate that glutamate signaling needs to be inhibited during LTM consolidation.

To identify *Eaat1*, we screened various genes regulating glial physiology for altered expression during LTM formation and found that expression of *Eaat1* and *crammer* increase after spaced training. As *Eaat1*, but not *crammer*, is expressed exclusively in glia (Soustelle et al., 2002), we focused on *Eaat1* as a likely Repo-regulated gene. Indeed, spaced-training-induced increases in EAAT1 depend on Repo and Klg activity. Interestingly, expression of the glial gene, *genderblind*, which encodes another glial glutamate transporter (Grosjean et al., 2008), required Repo activity for expression, but was not affected by spaced training, suggesting that other transcriptional regulatory factors besides Repo are likely necessary to differentially regulate genes required for memory consolidation from those required for other glial functions.

Because we only screened selected genes, it is possible that Repo induces the expression of other unidentified genes after spaced training. However, somewhat unexpectedly, we found that overexpression of *Eaat1* alone in glial cells is sufficient to rescue the LTM defects of *klg* and *repo* mutants. This indicates that the major function of the Klg/Repo signaling pathway is to induce glial expression of *Eaat1*. It further suggests that one function of astrocytes is to decrease glutamate signaling during LTM consolidation.

Combined with results from previous studies, our work identifies a putative pathway linking neuronal activity to glial inhibition of glutamate signaling. In flies, the homophilic cell adhesion molecule, Klingon, is expressed in both neurons and glia, and needs to be expressed in both cell types for normal LTM (Matsuno et al., 2015). Repo expression normally increases after spaced training, whereas it fails to do so in *klg* mutants, indicating that Klg-mediated neuron-glia communication is necessary for this increase (Matsuno et al., 2015). Thus, we propose that spaced training increases neuronal activity, which induces signaling to glia via the cell adhesion molecule Klg. This results in increased Repo activity in glia, which increases *Eaat1* expression, and subsequently decreases glutamate signaling.

Previous work from various groups including ours has shown that glutamate signaling through NMDA-type receptors (NRs) is necessary for learning and memory. Overexpression of NRs in mice enhances learning and memory formation (Kalev-Zylinska et al., 2009, Tang et al., 1999), and we and others have shown that glial production of D-serine, a neuromodulator that functions as a coactivator of NRs, is necessary for short-lasting memory (Billard, 2013, Yamazaki et al., 2014). In our current study, we focus on glutamate activity specifically during memory consolidation, instead of during initial learning and memory formation. Considering our current findings with those of previous studies, we propose that NR-dependent glutamate signaling needs to be initially high, during formation of short-lasting memories, but low during a later phase where short-lasting memories are consolidated into LTM. This suggests that glia play at least two roles in memory. They produce D-serine that contributes to high NR activity during memory formation and also produce EAAT1 after learning, which functions to reduce glutamate signaling during memory consolidation.

Age-related impairments in *Drosophila* memory do not consist of a general decrease in all forms of learning and memory, but instead consist of decreases in two specific phases of memory, MTM and LTM (Mery, 2007, Tamura et al., 2003). Our results suggest that both these memory effects are caused by age-related glial dysfunction. Glia in young flies are able to produce sufficient amounts of D-serine for normal MTM, whereas D-serine amounts decrease 2-fold in aged flies (Yamazaki et al., 2014). This decrease is responsible for age-related impairments in 1-h memory, because increasing glial production of D-serine, or directly feeding of D-serine to aged flies, rescues this impairment. Likewise, glial dysfunction is also responsible for age-related impairments in LTM because aged glia are unable to inhibit glutamate signaling during consolidation. Thus, in contrast to young flies, aged flies are unable to modulate glutamate activity during learning and consolidation, leading to defects in the two memory phases.

Our model that EAAT1 inhibits glutamate activity during consolidation stems from EAAT1's role in clearing glutamate from synaptic sites and transporting it into astrocytes (Besson et al., 1999, Kanai, 1997, Takahashi et al., 1997). This model is consistent with several mammalian studies that demonstrated decreased expression of astrocytic glutamate transporters upon aging, with a consequent reduction of glutamate uptake (Pereira et al., 2017, Potier et al., 2010). Further supporting this model, we found that feeding flies memantine or MK801, NMDA receptor antagonists, after spaced training, restores normal LTM in klg mutants and restores LTM in aged flies to youthful levels. This effect requires feeding after training during the consolidation phase. We obtained similar results by feeding riluzole, a glutamate modulator, which decreases glutamate release and increases astrocytic glutamate uptake (Nagoshi et al., 2015). Riluzole has also been reported to ameliorate age-related cognitive decline in mammals (Pereira et al., 2014), suggesting that the mechanisms of AMI may be conserved between species. In contrast, we found that D-serine feeding, which rescues age-related declines in short-lasting (1-h) memory, does not improve declines in LTM, but rather attenuates it. This is consistent with our model wherein declines in short-lasting memory and LTM are caused by distinct or opposing mechanisms and glutamate signaling needs to be suppressed during consolidation. Somewhat unexpectedly, we also found that (s)-4C3HPG, the mGluR1 antagonist/mGluR2 agonist, also ameliorated age-related impairments in LTM. This result indicates that glutamate activity through both ionotropic and metabotropic glutamate receptors antagonizes memory consolidation.

Currently, we are uncertain why glutamate signaling needs to be inhibited during consolidation, but a previous study has shown that Mg^2+^ block mutations in NMDA-type glutamate receptors (NRs) cause specific defects in LTM in *Drosophila* (Miyashita et al., 2012). Although Mg^2+^ block mutations have various effects, one effect is to increase NR activity. Increased NR activity results in increased activity of dCREB2b, an inhibitory isoform of CREB. CREB-dependent gene expression is required during consolidation of LTM (Hirano et al., 2016), suggesting that consolidation may be preferentially sensitive to NR activity.

Alternatively, it is possible that neuronal activity needs to be inhibited globally during memory consolidation. Sleep is known to be important for LTM. Sleep deprivation during consolidation prevents LTM formation (Ganguly-Fitzgerald et al., 2006), whereas artificially inducing sleep after training has been reported to improve LTM (Donlea et al., 2011). Thus we also suggest a second possibility that inhibition of glutamate signaling after spaced training may be a brain-wide phenomenon that promotes consolidation by inducing the organism to sleep. Thus far, we have not detected gross alterations in sleep duration in *klg* and *repo* mutants, although this does not preclude minor disruptions in sleep quality that may not be detectable by motion-based sleep assays. Finally, we envision a third possibility wherein neuronal inhibition may be required as a neuroprotective mechanism (Lewerenz and Maher, 2015) that may be necessary to prevent cell death in neurons that were extensively stimulated during spaced training.

Mapping the glutamatergic neurons whose activity is inhibited during consolidation will be of great interest in the future. As aversive olfactory memories are formed and stored in the *Drosophila* MBs (Heisenberg, 2003), it is possible that specific glutamatergic MB output neurons (MBONs) are inhibited during consolidation. Several glutamatergic MBONs are involved in feedback networks with the lobes of the MBs (Aso et al., 2014a, Aso et al., 2014b), suggesting that altering the activity of these neurons may modulate memory consolidation and memory-associated behavioral responses.

### Limitations of the Study

In our study, we demonstrate that increased expression of Eaat1 is required for LTM consolidation. Based on numerous results from other groups, we hypothesized that Eaat1 functions to reduce glutamate signaling, and we provide support for this model by demonstrating that pharmacological inhibition of glutamate signaling during consolidation improves LTM under various conditions. However, due to technical limitations, we have not been able to actually measure glutamate concentrations at synapses during memory consolidation and thus do not know where and how much glutamate signaling has to be inhibited for optimal LTM consolidation.

## Methods

All methods can be found in the accompanying Transparent Methods supplemental file.

## Supplemental Information

Document S1. Transparent Methods and Figures S1–S4
