# Supplementary material for: Inhibiting Glutamate Activity during Consolidation Suppresses Age-Related Long-Term Memory Impairment in Drosophila
Source: iScience. 2019 Apr 13;15:55–65. doi: 10.1016/j.isci.2019.04.014 (PMC6487374; doi:10.1016/j.isci.2019.04.014)
Supplement: Document S1. Transparent Methods and Figures S1–S4 [file mmc1.pdf]

ISCI, Volume *15*

Supplemental Information

Inhibiting Glutamate Activity during Consolidation Suppresses Age-Related Long-Term Memory Impairment in *Drosophila*

Motomi Matsuno, Junjiro Horiuchi, Kyoko Ofusa, Tomoko Masuda, and Minoru Saitoe

Supplemental Information

Inhibiting glutamate activity during consolidation suppresses age-related long-term memory impairment in *Drosophila*

Motomi Matsuno, Junjiro Horiuchi, Kyoko Ofusa, Tomoko Masuda, Minoru Saitoe

Four Supplemental Figures, Transparent Methods and Supplemental References.

Figure S1

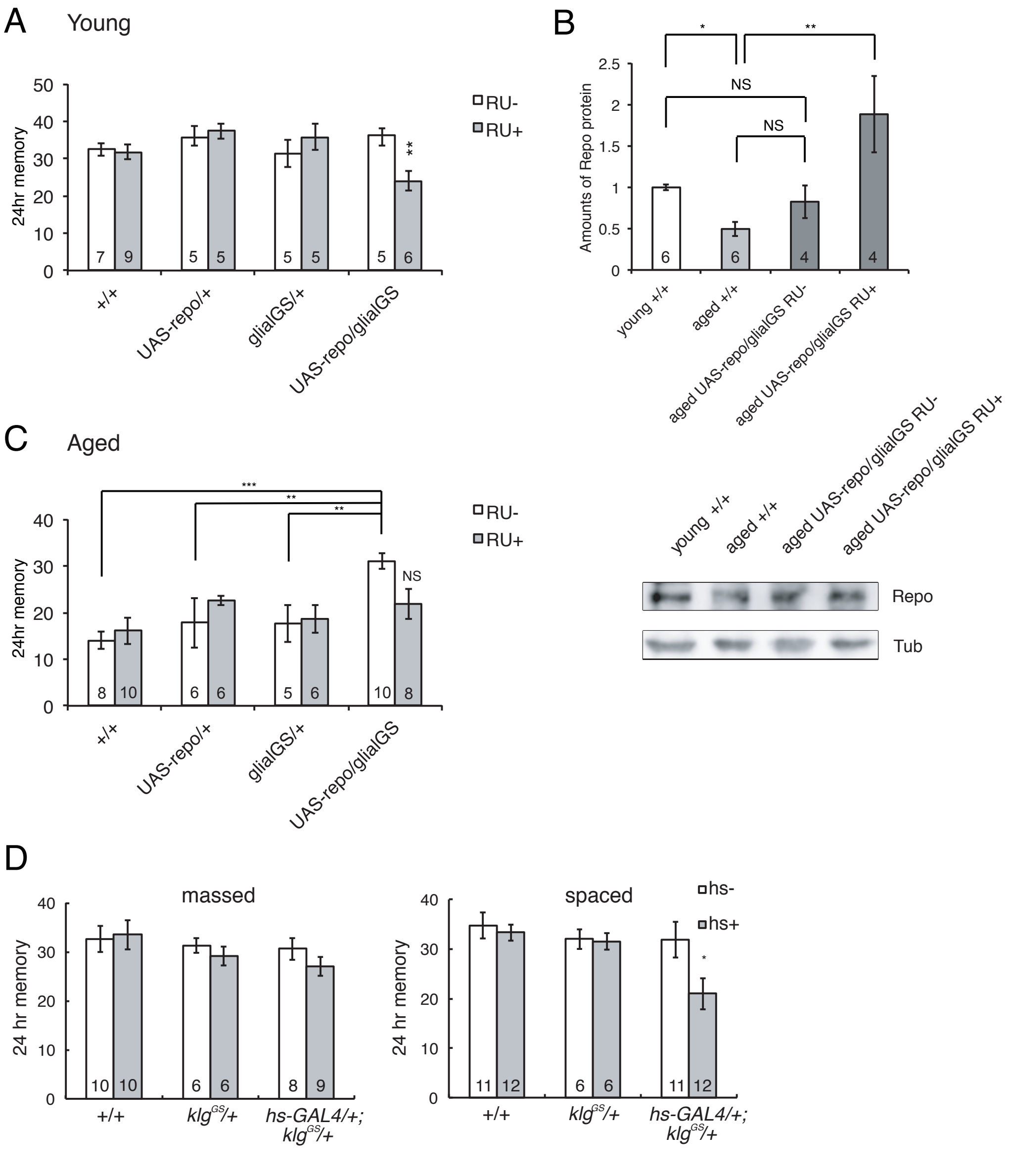
A
Young
24hr memory
50
40
30
20
10
0
RU-
RU+
**
7 9
5 5
5 5
5 6
+/+
UAS-repo/+
glialGS/+
UAS-repo/glialGS
B
*
**
NS
NS
Amounts of Repo protein
2.5
2
1.5
1
0.5
0
6
6
4
4
young +/+
aged +/+
aged UAS-repo/glialGS RU-
aged UAS-repo/glialGS RU+
C
Aged
***
**
**
24hr memory
40
30
20
10
0
RU-
RU+
NS
8 10
6 6
5 6
10 8
+/+
UAS-repo/+
glialGS/+
UAS-repo/glialGS
young +/+
aged +/+
aged UAS-repo/glialGS RU-
aged UAS-repo/glialGS RU+
Repo
Tub
D
massed
24 hr memory
40
30
20
10
0
10 10
6 6
8 9
+/+
klg^GS/+
hs-GAL4/+; klg^GS/+
spaced
hs-
hs+
24 hr memory
40
30
20
10
0
*
11 12
6 6
11 12
+/+
klg^GS/+
hs-GAL4/+; klg^GS/+

Figure S1. Overexpression of *repo* impairs LTM. Related to Figure 1.

(A) LTM, 24 hr memory after spaced training, was impaired in *glial-GS/UAS-repo* flies by feeding RU486 for 3 days prior to experiments. **p<0.01 compare to RU- control.

(B) Repo amount was tend to increase by leaky expression of repo transgene from glial-GS driver. Kruskal-Wallis test indicates significant differences between samples. *p < 0.05 and **p < 0.01 compare to aged +/+ flies. Bottom, Representative Western blot showing amounts of Repo in heads extract from young and aged wild-type (+/+), aged RU- *UAS-repo/glialGS* and aged RU+ *UAS-repo/glialGS*. α-Tubulin (Tub) amounts are shown for comparison.

(C) LTM was also declined in RU486 fed aged *glial-GS/UAS-repo* flies as compare to RU486 non-fed flies. *glial-GS/UAS-repo* rescues LTM-AMI. **p < 0.01 and ***p < 0.001 compare to *glial-GS/UAS-repo* flies.

(D) Left, Klg overexpression did not affect ARM, 24 hr memory after massed training. The $klg^{GS10439}$ mutation (klg^{GS}) results from an insertion of a P-UAS transposon 75 bp upstream of the proposed transcription start site (Seong et al. 2001) so that hs-GAL4 driver induces expression of *klg* transgene upon heat-shock (37℃ for 15 min).

Right, *klg* overexpression disrupted LTM, 24 hr memory after spaced training. Deleterious effect to LTM was not seen with hs-GAL4 upon heat-shock (Matsuno et al., 2009). *p < 0.05 compare to hs- flies. Data are represented as means ± SEMs.

Figure S2

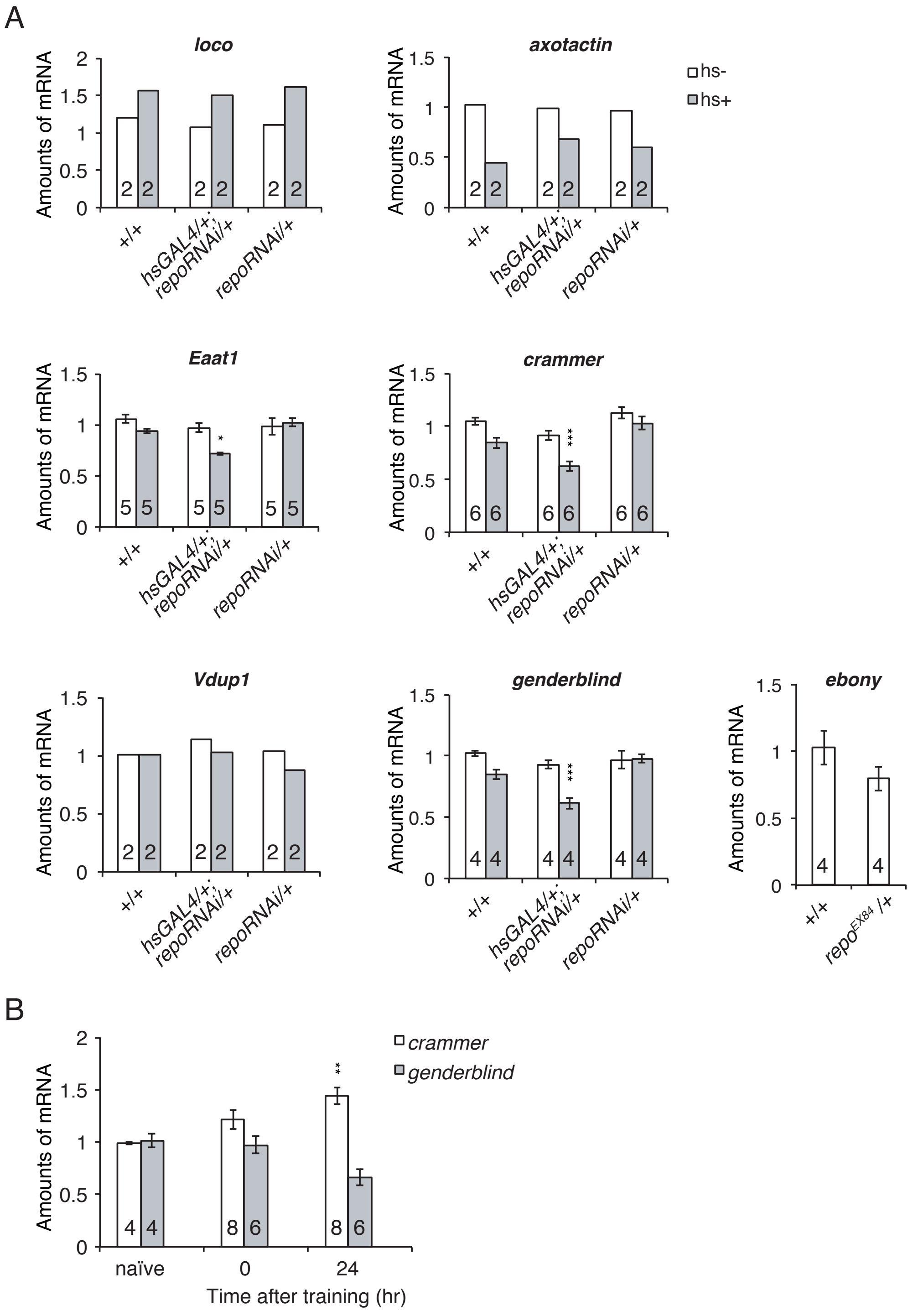

Figure S2. Brief screenings of Repo downstream genes. Related to Figure 2

(A) Relative amount of mRNA of seven candidate genes, *genderblind*, *crammer* and

Eaat1 expressions were reduced by inducing knock down of *repo* or *repo* mutation (*repo*^{EX84}). Some genes, including loco, axotaxin, and Vdup1 were only analyzed twice before being discarded as unlikely to be regulated by Repo. Because of this small sample size, we cannot conclude that expression of these genes is not affected by Repo knockdown.

(B) Changes in expression of candidate genes after spaced training. The increase in *crammer* becomes significant 24hr after training. **p<0.01 compared with naïve. Data are represented as means ± SEMs.

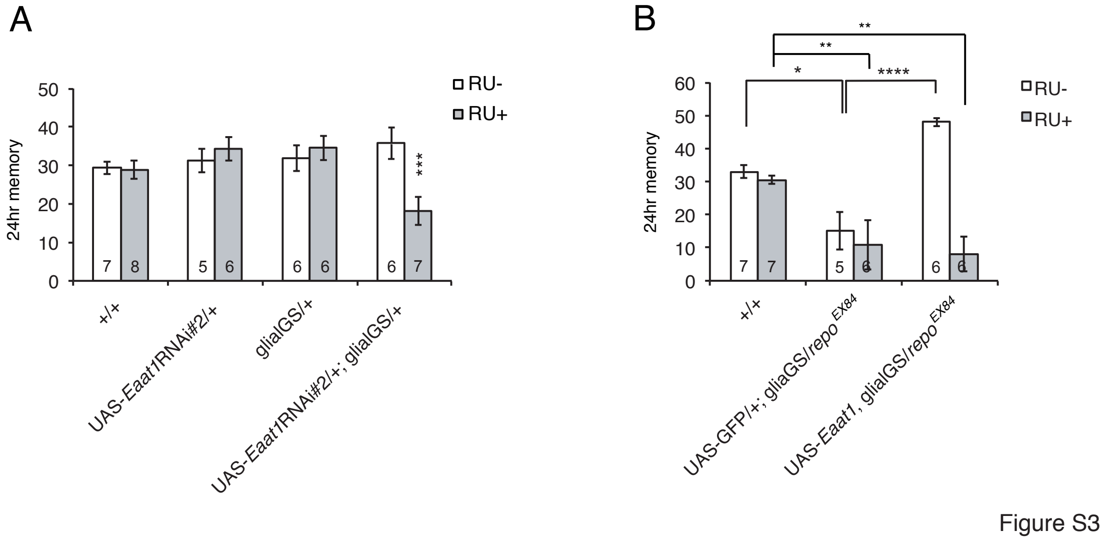

Figure S3 EAAT1 is required for LTM and functions downstream of Repo. Related to Figure 2.

(A) Acute inhibition of EAAT1 specifically disrupts LTM. The *glial-GS* driver was used to overexpress *Eaat1* RNAi#2 upon RU feeding in a *UAS-Eaat1 RNAi#2/+;glial-GS-GAL4/+* line. RU486 was fed to flies from 3 days before training until testing. ***p < 0.001 compared to RU- controls.

(B) Leaky expression of *Eaat1* in glial cells rescues LTM defects of *repo* mutant. Weak

glial expression of *Eaat1* (*UAS-Eaat1, glialGS/repo*^{EX84}) is sufficient to rescue these defects, while expression of an unrelated protein, GFP (*UAS-GFP/+; glialGS/ repo*^{EX84}) does not. *p<0.05, **p<0.01 and ****p<0.0001. Data are represented as means ± SEMs.

Figure S4

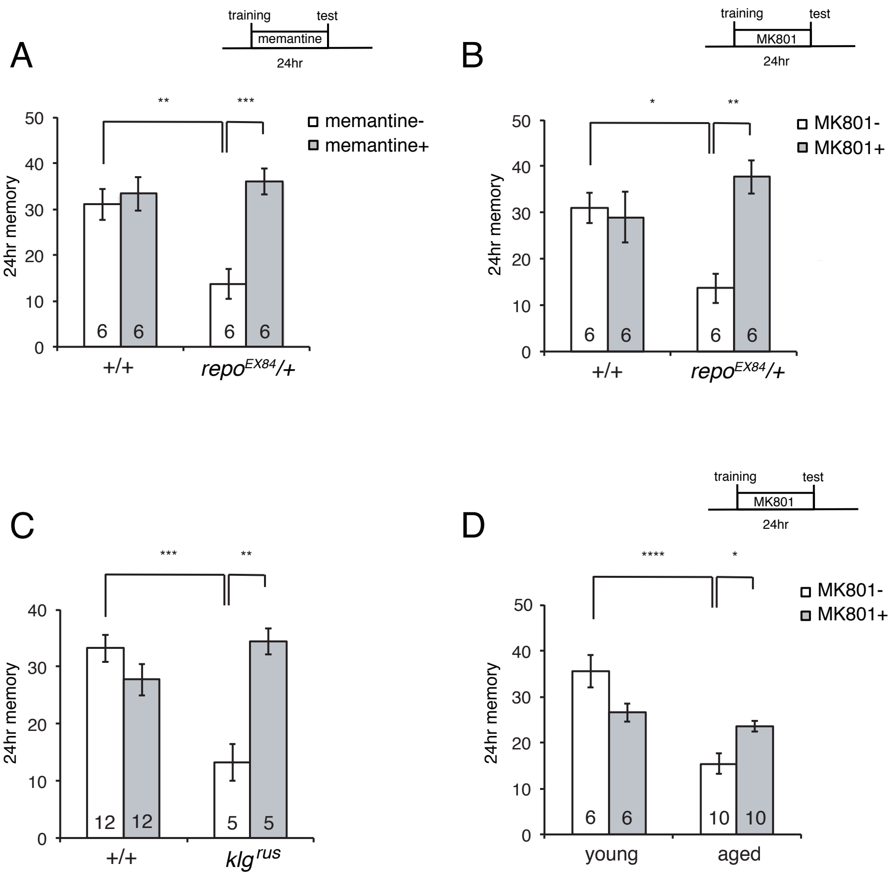

Figure S4 Inhibiting glutamate activity during consolidation rescues LTM in *klg* mutants, *repo* mutants and aged flies. Related to Figure 4.

(A) Feeding flies memantine (20ug/ml), an NMDAR antagonist, after spaced training significantly rescues LTM in *repo* mutants. **p<0.01 and ***p<0.001.

(B) Feeding flies 0.1mg/ml MK801, a NMDAR antagonist, immediately after spaced training significantly rescues the LTM in *repo* mutants. *p<0.05 and **p<0.01.

(C, D) Feeding flies 0.1mg/ml MK801 immediately after spaced training significantly

rescues the LTM in both *klg* mutants (C) and aged flies (D). *$p<0.05$, **$p<0.01$, ***$p<0.001$ and ****$p<0.0001$. Data are represented as means ± SEMs.

Transparent Methods

Fly stocks and genetics:

Flies were raised under a 12h:12h, light: dark cycle at 25 degrees and 60% humidity. All flies used in this study were outcrossed to our wild-type line, w(CS10), for at least six generations. Approximately 100 flies were raised in food vials and transferred to fresh vials every 2 or 3 days. *hs-GAL4*, *UAS-klg RNAi* (klg^{36162}), klg^{rus}, $klg^{GS10439}$ and klg^{E226} (Matsuno et al., 2009), $repo^{EX84}$, *UAS-repo RNAi* ($repo^{TRIP.\ JF02974}$), $UAS\text{-}repo^{DN}$ ($repo^{\Delta AD302}$), *UAS-repo-myc*, the glial-geneswitch line, GSG3285-1 and *UAS-GFP* (Matsuno et al., 2015); MZ0709 (Doherty et al., 2009), *Eaat1-GAL4* and *UAS-Eaat1 RNAi* (*UAS-Eaat1-IRII*) (Rival et al., 2004) have all been described previously. $UAS\text{-}Eaat1^{\#8202}$ was obtained from the Bloomington Stock Center (Indiana University), and UAS-*Eaat1* RNAi#2 ($Eaat1^{109401}$) was obtained from the Vienna Drosophila RNAi Center.

Drug treatments:

For RU486 treatment, RU486 (mifepristone, Sigma) was dissolved in ethanol, and mixed with fly food to a final concentration of 1% ethanol and 0.5 mM RU. Flies were fed RU486 for indicated periods of time. For riluzole (Sigma), memantine (Sigma), MK801 (TOCRIS), (s)-4C3HPG (TOCRIS), and D-Serine (Wako) treatment, flies were transferred to vials containing strips of Whatman filter paper soaked with indicated concentrations of each drugs and 5% sucrose. All drugs have been previously shown to affect neuronal or behavioral activity in *Drosophila* (see references), and effective concentrations were chosen from these references. For D-ser, the only drug that did not affect LTM-AMI, the 1 mM concentration used has previously been shown to ameliorate MTM-AMI (Yamazaki et al., 2014).

Learning and memory assays:

Standard single-cycle conditioning was performed as previously described (Tully et al.,

1990). 100 flies were trained to the CS+ and CS- odors, with the CS+ odor paired with electric pulses. 3-octanol [OCT] and 4-methylcyclohexanol [MCH]) were used for conditioned stimuli (CS), and 1.5 s pulses of 60V DC electric shocks were used as the unconditioned stimuli (US). A performance index (PI) was calculated so that a 50:50 distribution (no memory) yielded a PI of zero and a 0:100 distribution away from the CS+ yielded a PI of 100.

Spaced and massed training sessions were also performed as described previously (Tully et al., 1994). Spaced training consists of 10 single-cycle training sessions, with a 15 min rest intervals. Massed training consists of 10 cycles of training without any rest intervals. After training, flies were stored in an 18 degrees incubator on a 12h:12h, light: dark cycle until testing. A performance index was measured 24hr after spaced or massed training.

Quantification of transcripts by quantitative PCR:

Total RNA from *Drosophila* heads was extracted with TRIzol reagent (Invitrogen) and cDNA was synthesized using RevTraAce (ToYoBo) as described previously (Matsuno et al., 2015). Transcript amounts were quantified using real-time PCR (model 7500; Applied Biosystems). GAPDH2 and rp49 were used for normalization. Primers used were as follows:

klg-F: 5’-GGAGTGCAAGGGATCTGGTA-3’

klg-R: 5’-GCTCCAGCTTCTCCAGTGTC-3’

repo-F: 5’-ACCATTTGGATCATGGCAGT-3’

repo-R: 5’-AACACATCTCATCGGGCTTC-3’

Eaat1-F: 5’-CCTCATCATAGCCGTCGATT-3’

Eaat1-R: 5’-GGCCAAATCATTCTTCGACA-3’

GAPDH2-F: 5’-GCGGTAGAATGGGGTGAGAC-3’

GAPDH2-R:	5’-TGAAGAGCGAAAACAGTAGC-3’
rp49-F:	5’-AGCATACAGGCCCAAGATCGTG-3’
rp49-R:	5’-CGACAGCTTAGCATATCGATCC-3’
loco-F:	5’-GACTTACTCCGCGTCTTTCG-3’
loco-R:	5’-GCGGATTGGATCTAGACGAG-3’
axo-F:	5’-ATTGCTTTGGCACCCACTAC-3’
axo-R:	5’-TAATACGCCTGGTGGAGGAG-3’
cer-F:	5’-CCTTCTCGAACTTCCGATTG-3’
cer-R:	5’-CCCTGGTTTCAGATGAGGAG-3’
Vdup1-F:	5’-CTGACCTCCACCAACACAGA-3’
Vdup1-R:	5’-GTGATGAATCCCATCGACCT
gb-F:	5’-AAGAAGGGTTCCAGCACGTA-3’
gb-R:	5’-CGGCTTTCCTGTATCTCTGG-3’
e-F:	5’-GACATTATTGTGGCTAGCTTCTATAACAAG-3’
e-R:	5’-CGCTGTAGTCGGTTCTCAAAACT-3’

Western blotting:

Monoclonal antibody against Repo (1:20, 8D12 from Hybridoma bank) was used for westernblot of Repo. Mouse anti-α-tubulin antibody (1:1000, #DM1a from Seikagaku Kogyo) was used for normalization. Head extracts were made in homogenization buffer [25mM HEPES, 100mM NaCl, 1mM $MgCl_2$, 1mM $CaCl_2$, 0.2% Trion X-100, 0.2% Nonidet P-40 and protease inhibitors (Roche)]. Signals were detected using HRP conjugated secondary antibodies and ECL blotting reagents (GE Healthcare).

Statistics:

All data are expressed as mean ± SEM. Sample size are indicated in each bar in all graphs. Student’s t-test was used to evaluate the statistical significance between two

data sets. For multiple comparisons, one-way, two-way ANOVA or Kruskal-Wallis test was used, followed by post hoc analyses using Prism software (GraphPad Software, Inc., La Jolla, CA, USA).

Supplemental References

Doherty, J., Logan, M.A., Tasdemir, O.E., and Freeman, M.R. (2009). Ensheathing glia function as phagocytes in the adult Drosophila brain. J Neurosci *29*, 4768-4781.

Matsuno, M., Horiuchi, J., Tully, T., and Saitoe, M. (2009). The Drosophila cell adhesion molecule klingon is required for long-term memory formation and is regulated by Notch. Proc Natl Acad Sci U S A *106*, 310-315.

Matsuno, M., Horiuchi, J., Yuasa, Y., Ofusa, K., Miyashita, T., Masuda, T., and Saitoe, M. (2015). Long-term memory formation in Drosophila requires training-dependent glial transcription. J Neurosci *35*, 5557-5565.

Rival, T., Soustelle, L., Strambi, C., Besson, M.T., Iche, M., and Birman, S. (2004). Decreasing glutamate buffering capacity triggers oxidative stress and neuropil degeneration in the Drosophila brain. Curr Biol *14*, 599-605.

Tully, T., Boynton, S., Brandes, C., Dura, J.M., Mihalek, R., Preat, T., and Villella, A. (1990). Genetic dissection of memory formation in Drosophila melanogaster. Cold Spring Harb Symp Quant Biol *55*, 203-211.

Tully, T., Preat, T., Boynton, S.C., and Del Vecchio, M. (1994). Genetic dissection of consolidated memory in Drosophila. Cell *79*, 35-47.

Yamazaki, D., Horiuchi, J., Ueno, K., Ueno, T., Saeki, S., Matsuno, M., Naganos, S., Miyashita, T., Hirano, Y., Nishikawa, H., *et al.* (2014). Glial dysfunction causes age-related memory impairment in Drosophila. Neuron *84*, 753-763.
